# Slice-level vs. volumetric radiomics for molecular subtyping of breast cancer: impact of patient-level data partitioning on model performance

**DOI:** 10.3389/fradi.2026.1915426

**Published:** 2026-08-31

**Authors:** Laila El Jiani, Zouheir Banou, Fatima Zahra Alaoui, Hasnae Sakhi, El Habib Benlahmar, Sanaa El Filali

**Affiliations:** 1 Laboratory of Artificial Intelligence and Systems, Faculty of Science Ben M’sick, Hassan II University, Casablanca, Morocco; 2 Laboratory of Information Processing and Modeling, Faculty of Science Ben M’sick, Hassan II University, Casablanca, Morocco

**Keywords:** 2D vs. 3D radiomic features, Basal-like, breast cancer, Luminal A, molecular subtype classification, patient-level leakage prevention, radiomics, TCGA-BRCA

## Abstract

**Introduction:**

Molecular subtyping of breast cancer, particularly the discrimination between Luminal A and Basal-like tumours, is critical for guiding treatment decisions and establishing prognosis. Radiomics offers a promising complementary, non-invasive imaging biomarker to support biopsy-based subtyping; however, two methodological gaps limit the validity of existing studies: the absence of controlled 2D-vs.-3D feature comparisons, and the systematic underreporting of patient-level data leakage in multi-slice pipelines.

**Methods:**

To address both limitations, this study benchmarks slice-level (2D) and volumetric (3D) radiomic pipelines for the classification of Luminal A vs. Basal-like tumours on the TCGA-BRCA cohort, evaluating seven classifiers combined with two feature selection strategies under a Stratified Group *k*-Fold (*k* = 5) cross-validation scheme that enforces strict patient-level isolation.

**Results:**

The 3D pipeline achieved a mean AUC of 0.682 vs. 0.631 for the 2D pipeline. In the 3D configuration, the Support Vector Machine with SelectKBest-MI feature selection attained the highest performance (AUC = 0.768 ± 0.066), while the 2D configuration was led by Logistic Regression (AUC = 0.769 ± 0.109), albeit with substantially higher fold-to-fold variability. Six out of seven classifiers yielded higher AUC scores under the 3D configuration, although a paired significance test on fold-level AUC values did not reach statistical significance (*p* = 0.313).

**Discussion:**

These results establish a reproducible, leakage-free baseline for non-invasive breast cancer subtype classification through radiomics, with 3D radiomics offering improved robustness rather than superior peak performance. Results are derived from a single cohort (TCGA-BRCA) and external validation is required before clinical generalizability can be claimed.

## 1 Introduction

Breast cancer is the most frequently diagnosed malignancy in women worldwide, accounting for approximately 2.3 million new cases and 685,000 deaths in 2020 [1]. Its clinical management is driven by molecular subtype classification, Luminal A, Luminal B, HER2-enriched, and Basal-like, established through gene expression profiling and associated with fundamentally different prognoses, therapeutic responses, and recurrence risks [2]. Luminal A tumours, characterised by high oestrogen receptor (ER) expression and low proliferative activity, carry the most favourable prognosis and respond well to endocrine therapy alone. Basal-like tumours, conversely, are ER${}^{-}$, PR${}^{-}$, and HER2${}^{-}$, exhibit high genomic instability and rapid proliferation, and are associated with the worst prognosis and highest recurrence rates [3]. In this study, the term *Basal-like* refers to the PAM50 transcriptomic subtype label as annotated in TCGA-BRCA, which correlates strongly with, but is not clinically synonymous with, triple-negative breast cancer (TNBC) defined by IHC receptor status; the two terms are not used interchangeably here.

The current gold standard for subtype determination relies on immunohistochemistry (IHC) applied to core needle biopsy specimens, combined with fluorescence in situ hybridisation (FISH) for HER2 assessment [4]. While highly reliable under optimal conditions, this paradigm carries well-documented limitations: biopsy is invasive, subject to spatial sampling bias [only ∼78% concordance between biopsy and surgical specimen receptor status [5]], carries procedural risk (2%–15% haematoma incidence [6]), and cannot characterise whole-tumour spatial heterogeneity, a critical limitation for Basal-like tumours, which exhibit high subclonal diversity [7, 8]. Radiomics, the high-throughput extraction of quantitative features from medical images, offers a non-invasive alternative that encodes tumour morphology, texture, and heterogeneity from routine imaging without additional patient contact [9, 10].

Despite growing interest in radiomics-based subtype classification, three methodological gaps persist in the literature. First, multi-slice 2D radiomics pipelines are frequently affected by patient-level leakage, assigning slices from the same patient to both training and test sets, inflating AUC by 0.08–0.15 [11] and producing results non-reproducible in prospective deployment [12]. Second, direct controlled comparisons of 2D slice-level and 3D volumetric configurations under identical classifiers and validation conditions remain rare, making it difficult to assess when the added cost of full volumetric segmentation is justified by performance gains [13]. Third, reported performance metrics often lack honest uncertainty quantification across folds, obscuring the true variability of results in small, imbalanced cohorts [14].

The purpose of this paper is to address the three mentioned gaps with three contributions. First, it provides an automated patient stratified cross- validation process free of leakage using programmatically verified patient isolation; Second, it guarantees that every classifier and both feature selection methods were tested for 7 classifiers in 2D and 3D (on TCGA-BRCA, *n* = 88, Luminal A vs Basal-like); Finally, it provides an exact reporting of the mean performance metrics and the variability in fold-to-fold performance. The results show that the 3D configuration achieves a higher mean AUC (0.682 vs. 0.631 for 2D) with better fold-to-fold stability (best std 0.066 vs. 0.109), while both configurations reach comparable peak performance (AUC = 0.769 for 2D and AUC = 0.768 for 3D) through different reliability profiles.

## 2 Related work

### 2.1 Radiomics for breast cancer molecular subtype classification

Radiogenomics is a concept that links imaging phenotypes with molecular characteristics. In breast cancer, Mazurowski et al. have described radiogenomic relationships in the form of DCE-MRI features correlated with gene expression profiles of breast invasive ductal carcinoma patients within the TCGA-BRCA cohort [15]. Their data has also shown that MRI enhancement dynamics were correlated with the Luminal B breast cancer subtype, providing initial evidence that imaging phenotypes reflect molecular states associated with cancer. Similarly, Grimm et al. also found kinetic (dynamic) features from MRI correlated with the intrinsic subtypes of breast cancer, achieving an AUC of 0.62–0.70 for separating Luminal from Non-Luminal subtypes [16]. Saha et al. [17] also transitioned from unsupervised classification of imaging features to supervised classification of Luminal A and Triple-Negative breast cancer using a radiomic signature based on 529 imaging features derived from DCE-MRI. Using Random Forest classification, they achieved an AUC of 0.71 for separating the two subtypes. The authors showed that the most discriminative features for Basal-like identification were entropy, energy, and GLCM-based descriptors. Subsequently, Li et al. demonstrated similar discrimination using multiparametric MRI (T2+DCE), achieving an AUC of 0.74 for predicting four subtypes of breast cancer while noting that T2-weighted texture features had better scanner stability than DCE kinetic features.

Studies that exceed AUC = 0.85 consistently rely on multiparametric MRI sequences (DCE, DWI, T2) unavailable in CT-only settings, or incorporate deep learning features trained on large datasets (n>500) [18, 19]. Leithner et al. achieved AUC = 0.86 for Triple-Negative vs. other subtypes using multiparametric MRI with a multi-layer perceptron, while DCE subregion analysis has reported AUC values up to 0.92 for Luminal vs. HER2 discrimination by modelling intratumoural kinetic heterogeneity [20]. For single-sequence MRI radiomics—the configuration most directly comparable to our work is Saha et al. [17] reported AUC = 0.71 on TCGA-BRCA using DCE-MRI and Random Forest, establishing the most relevant baseline for our study.

### 2.2 2D vs. 3D radiomics: evidence and trade-offs

According to the Image Biomarker Standardization Initiative [21], the extraction of three-dimensional characteristics of objects is recognized as the standard method for achieving reproducible radiomics, since two-dimensional characteristics depend on the selection of slices and the angle at which they are obtained. Three-dimensional features represent the complete spatial heterogeneity of a tumour through all three axes and include texture and morphology variations; which are not present on any two-dimensional slice. In [22], authors conducted an investigation with lung cancer patients and found that volumetric features of the shape of a tumour provide prognostic value independent of measurements from single slices. The transition from two-dimensional to three-dimensional radiomics has been associated with an increase in area under the receiver operating characteristic curve between 0.05 and 0.10 in brain tumour grade studies due to the added global information about the morphology of the tumour as a whole [23].

Despite these theoretical advantages, 2D slice-level extraction has practical merits that justify its continued use in clinical settings. Full volumetric segmentation is time-consuming and operator-dependent, whereas radiologists routin annotate representative slices in diagnostic workflows [24, 25]. From a machine learning perspective, 2D multi-slice extraction provides a larger effective training set per patient, a non-trivial advantage when the cohort is small [26]. In the multi-slice setting, each patient contributes multiple observations, increasing the diversity of training examples within each fold, at the cost of introducing within-patient dependencies that must be addressed through appropriate patient-level validation.

Direct controlled comparisons of 2D and 3D pipelines on the same dataset under identical classifiers, feature selection methods, and validation strategies remain rare in the breast cancer subtype literature [17–20, 25–27]. The absence of such systematic comparisons makes it difficult to assess when the added segmentation cost of 3D extraction is justified by performance gains—a clinically relevant question that this study directly addresses.

### 2.3 Data leakage in radiomics validation

Data leakage is a pervasive problem in radiomics, with particularly severe consequences in multi-slice 2D studies [12]. When standard k-fold cross-validation is applied without patient-level grouping, slices from the same patient appear in both training and test sets, allowing the model to recognise patients it has effectively already seen [26, 28]. Demircioğlu et al. [11] demonstrated that incorrect feature selection placement before cross-validation inflates AUC by up to 0.15 in AUC-ROC, 0.29 in AUC-F1, and 0.17 in accuracy, across ten publicly available radiomics datasets. Approximately 10%–15% of published radiomics studies using only cross-validation misapply feature selection, exacerbating the reproducibility problem in the field.

A second, often overlooked form of leakage occurs when feature selection is performed on the entire dataset before cross-validation, allowing test-set label information to influence feature ranking [12, 28]. Both forms of leakage produce AUC estimates that do not reflect genuine generalisation to unseen patients, the clinically relevant target [14, 29]. Stratified Group K-Fold cross-validation, which simultaneously enforces patient-level grouping and class stratification, is increasingly recognised as the reference standard for multi-slice radiomics validation. This study implements StratifiedGroupKFold with programmatic patient isolation verification and strictly enforces feature selection within each training fold.

Table 1 summarises the positioning of this study relative to key related works across six criteria. To our knowledge, no prior study combines leakage-free patient-stratified validation with a systematic 2D vs. 3D comparison for breast cancer molecular subtype classification under identical experimental conditions.

**Table 1:** Positioning of this study relative to key related works.

Study	Modality	Task	n	Method	AUC	2D/3D	Leak-free
Li et al. [27]	DCE-MRI	ER/PR/HER2/TN	91	LDA + LOO-CV	0.89	3D	Partial
Leithner et al. [19]	mpMRI (DCE+DWI)	LumA vs TN	91	MLP-ANN	0.86	2D	N.R.
Romeo et al. [18]	PET/MRI	TNBC vs others	86	SVM + LASSO	0.887	3D	N.R.
Huang et al. [20]	DCE-MRI	4-class	377	Logistic Reg.	0.786	3D	N.R.
Lee et al. [26]	MRI (3T)	Subtypes + Ki67	291	Random Forest	0.80	3D	N.R.
Saha et al. [17]	DCE-MRI	LumA vs TNBC	922	Random Forest	0.71	3D	Partial
**This study (2D)**	**MRI**	**LumA vs Basal**	**88**	**7 classifiers**	**0.769**	**2D**	**Yes**
**This study (3D)**	**MRI**	**LumA vs Basal**	**88**	**7 classifiers**	**0.768**	**3D**	**Yes**

N.R., Not reported; mpMRI, multiparametric MRI; LOO-CV, Leave-one-out cross-validation; LDA, Linear Discriminant Analysis.

Bold rows indicate the results of the present study (2D and 3D configurations).

## 3 Methodology

**Figure 1:**
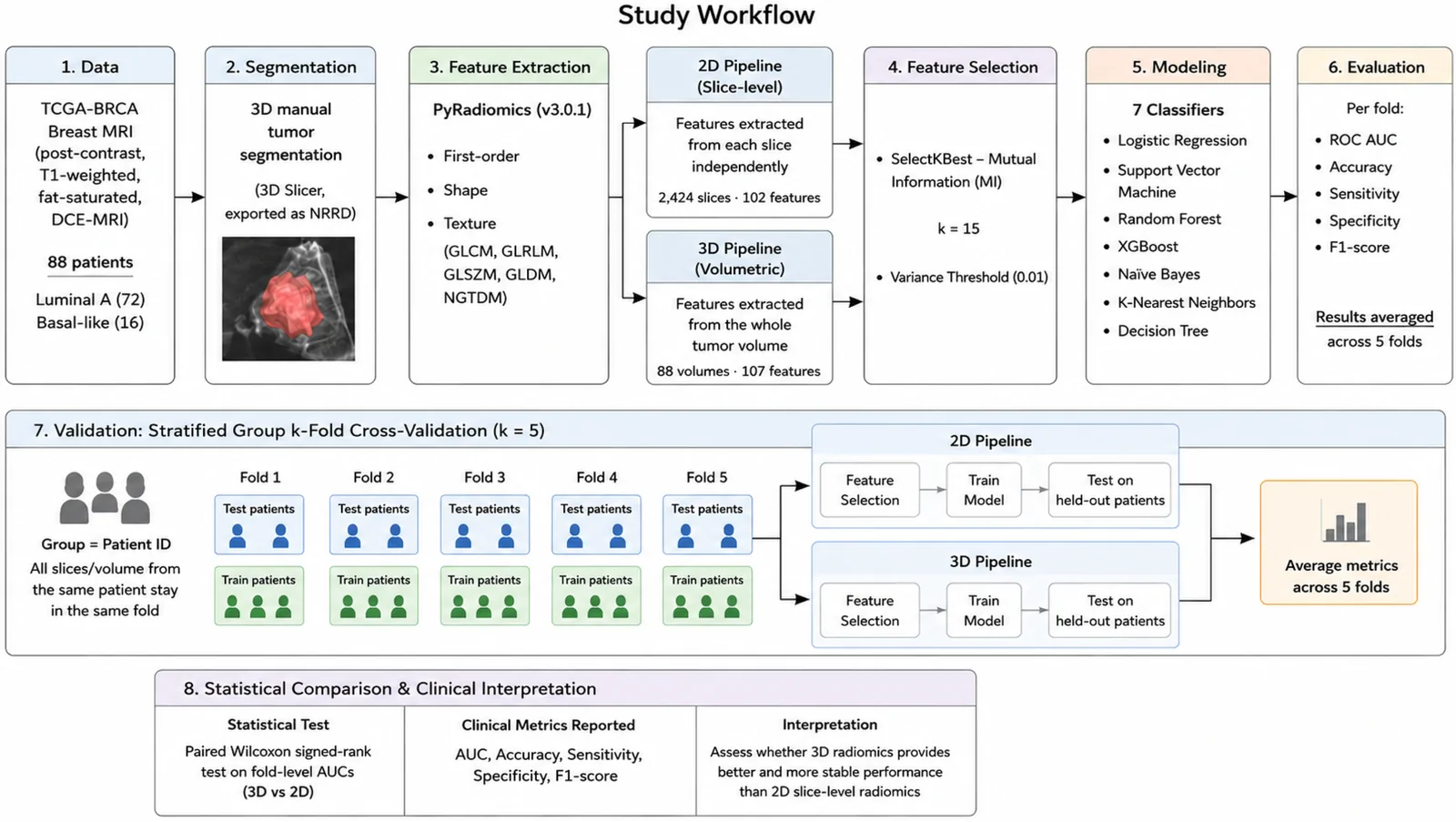
Overview of the study workflow, from patient selection through statistical comparison, illustrating the parallel 2D (slice-level) and 3D (volumetric) radiomics pipelines applied to the same segmented tumor volumes.

Figure 1 provides an overview of the complete study workflow, from patient selection to statistical comparison. The pipeline proceeds through eight stages: patient cohort selection, tumor segmentation, radiomics feature extraction, parallel 2D and 3D feature selection, classification, per-fold evaluation, patient-stratified cross-validation, and final statistical comparison between configurations. Each stage is detailed in the corresponding subsection below.

### 3.1 Dataset and patient cohort

We use the TCGA-BRCA cohort accessed via the GDC Data Portal [30], restricted to Luminal A (n=72) and Basal-like (n=16) patients with available imaging and PAM50 molecular subtype annotations (n=88 total). The class imbalance ratio is 4.5:1, addressed through class-weight balancing during classifier training. The 2D configuration yields 2,424 slice-level observations (mean 27.5 slices per patient) with 102 radiomics features after removing diagnostic columns; the 3D configuration yields 88 volumetric observations with 107 features. Table 2 summarises the dataset statistics per configuration. Missing values are imputed per-feature using the training-fold median to prevent information leakage from the test set.

**Table 2:** Dataset statistics per radiomics configuration.

Characteristic	2D Configuration	3D Configuration
Patients (n)	88	88
Luminal A/Basal-like	72 / 16	72 / 16
Class ratio	4.5:1	4.5:1
Total observations	2,424 slices	88 volumes
Radiomics features	102	107
Cross-validation	StratifiedGroupKFold (k=5)	StratifiedGroupKFold (k=5)
Patient isolation verified	Yes (programmatic)	Yes (structural)

### 3.2 Imaging data and tumor segmentation

Imaging data consist of T1-weighted, fat-saturated, dynamic contrast-enhanced (DCE) MRI, using the first post-contrast acquisition phase, obtained from the TCGA-BRCA collection on TCIA. As TCGA-BRCA aggregates data from multiple institutions, acquisition plane and scanner protocol vary by contributing site; this heterogeneity is acknowledged as a limitation (Section 5). Tumor regions of interest were manually segmented in 3D Slicer and exported as binary labelmaps in NRRD format (image–mask pairs). While automated and semi-automated segmentation approaches, including foundation models such as the Segment Anything Model, are increasingly explored for breast imaging [31], manual segmentation was adopted here to ensure high-fidelity tumor boundary delineation given the exploratory scope of this study.

Radiomic features were extracted from these NRRD volumes using PyRadiomics (v3.0.1) [32]. For the 3D configuration, features were computed directly on the full volumetric mask (force2D=False), with original voxel spacing preserved (no resampling) and a fixed bin width of 25 for gray-level discretization. For the 2D configuration, the same NRRD volumes were processed slice-by-slice (force2D=True): each axial slice containing tumor ROI was treated as an independent 2D observation, yielding the 2,424 slice-level samples described in Table 2.

### 3.3 Radiomics feature extraction

Radiomics features are extracted from MRI imaging data in both 2D and 3D configurations. The 2D configuration extracts features per axial slice, covering shape2D descriptors, first-order intensity statistics, and texture matrices (GLCM, GLRLM, GLSZM, NGTDM). The 3D configuration extracts features from the full tumour volume, additionally capturing volumetric shape descriptors including volume, surface area, sphericity, and elongation. All diagnostic columns generated during extraction (prefixed diagnostics_) are removed prior to analysis, as they encode pipeline metadata rather than tumour phenotypic information.

### 3.4 Feature selection

Two feature selection strategies are evaluated and applied strictly within each cross-validation fold on the training partition only:

- **SelectKBest with Mutual Information (SelectKBest-MI, k=15):** ranks features by their mutual information with the target label, capturing non-linear feature–label dependencies. The top 15 features per fold are selected.
- **Variance Threshold (threshold = 0.01):** removes quasi-constant features with near-zero variance, eliminating uninformative features that contribute noise without discriminative power.

Feature selection is performed exclusively within the training partition of each fold to prevent test-set label information from influencing feature ranking, a critical safeguard against the second form of leakage described in Section 2.3. Synthetic oversampling techniques such as SMOTE or ADASYN were not applied in this study. With only 16 Basal-like patients and 102–107 radiomics features, interpolation-based oversampling operates in a high-dimensional space with very few real neighbours per class, risking the generation of synthetic feature vectors that do not correspond to plausible tumour phenotypes—a known concern in small-n radiomics. Class-weight adjustment (class_weight=balanced, scale_pos_weight) was preferred as it penalizes misclassification of the minority class without fabricating feature vectors. A comparative evaluation of oversampling strategies is left for future work at larger cohort sizes.

### 3.5 Classification models

Seven supervised classifiers are evaluated to assess the robustness of the pipeline across different learning paradigms. Table 3 summarises the classifiers and their key hyperparameters. All classifiers that support class-weight adjustment are configured with class_weight=balanced; for XGBoost, scale_pos_weight is computed dynamically per training fold as the ratio of negative to positive samples.

**Table 3:** Classifiers and key hyperparameters.

Classifier	Key Hyperparameters	Imbalance Handling
Logistic Regression	solver = saga, max_iter = 2000	class_weight = balanced
Random Forest	n_estimators = 100, random_state = 42	class_weight = balanced
Decision Tree	random_state = 42	class_weight = balanced
SVM	kernel = rbf, probability = True	class_weight = balanced
Naive Bayes	GaussianNB (default)	N/A
KNN	n_neighbours = 5	N/A
XGBoost	n_estimators = 100, eval_metric=logloss	scale_pos_weight per fold

### 3.6 Validation strategy and leakage prevention

Stratified Group K-Fold cross-validation (k=5) assigns all slices from a given patient exclusively to either the training or the test partition, preventing patient-level leakage. The grouping variable is the patient identifier (patient_id). Stratification ensures that the Luminal A/Basal-like class ratio is preserved across folds. Patient isolation is verified programmatically at each fold: |train_groups ∩ test_groups| = 0 any violation triggers immediate pipeline interruption. For the 3D configuration, each patient contributes exactly one volume, making patient-level leakage structurally impossible; StratifiedGroupKFold is nonetheless applied for consistency. For the 2D configuration, slice-level predicted probabilities are aggregated to the patient level by averaging (mean-pooling) prior to AUC computation, ensuring a clinically equivalent, patient-level comparison with the 3D configuration. Performance is reported as mean ± std AUC, accuracy, and weighted F1 across five folds, using AUC as the primary metric. Inverted AUC values (< 0.5) are corrected by label flipping, consistent with standard practice.

## 4 Results

### 4.1 Full classification results

Tables 4, 5 report all 14 classifier–feature-selection combinations for the 2D and 3D configurations respectively, sorted by descending AUC. The best combination per configuration is highlighted in bold.

**Table 4:** 2D slice-level results, sorted by AUC.

Classifier	Feature selection	AUC	±Std	Acc	F1
**Log. Regression**	**SelectKBest (MI)**	**0.769**	**0.109**	**0.570**	**0.564**
Naive Bayes	Variance Threshold	0.741	0.148	0.481	0.465
Log. Regression	Variance Threshold	0.699	0.150	0.564	0.552
Naive Bayes	SelectKBest (MI)	0.648	0.181	0.619	0.535
SVM	Variance Threshold	0.634	0.099	0.598	0.587
XGBoost	SelectKBest (MI)	0.632	0.078	0.661	0.619
XGBoost	Variance Threshold	0.628	0.085	0.648	0.609
Random Forest	Variance Threshold	0.628	0.058	0.603	0.548
SVM	SelectKBest (MI)	0.615	0.074	0.628	0.615
KNN	Variance Threshold	0.590	0.101	0.619	0.580
Random Forest	SelectKBest (MI)	0.586	0.060	0.643	0.575
Decision Tree	SelectKBest (MI)	0.571	0.062	0.695	0.662
Decision Tree	Variance Threshold	0.563	0.050	0.657	0.635
KNN	SelectKBest (MI)	0.536	0.026	0.640	0.611
**Mean**		**0.631**	–	0.616	0.583

Best result in bold.

**Table 5:** 3D volumetric results, sorted by AUC.

Classifier	Feature selection	AUC	±Std	Acc	F1
**SVM**	**SelectKBest (MI)**	**0.768**	**0.066**	**0.783**	**0.753**
Log. Regression	Variance threshold	0.748	0.127	0.714	0.705
Naive Bayes	Variance threshold	0.727	0.157	0.495	0.443
Random forest	SelectKBest (MI)	0.698	0.101	0.702	0.613
XGBoost	SelectKBest (MI)	0.697	0.088	0.670	0.636
Naive Bayes	SelectKBest (MI)	0.691	0.173	0.668	0.611
SVM	Variance threshold	0.689	0.049	0.693	0.674
Decision tree	SelectKBest (MI)	0.684	0.124	0.767	0.743
Log. regression	SelectKBest (MI)	0.680	0.100	0.714	0.696
Random forest	Variance threshold	0.647	0.058	0.696	0.610
XGBoost	Variance threshold	0.647	0.142	0.652	0.612
KNN	Variance threshold	0.630	0.122	0.737	0.681
Decision Tree	Variance Threshold	0.628	0.113	0.718	0.689
KNN	SelectKBest (MI)	0.616	0.130	0.696	0.629
**Mean**		**0.682**	–	0.693	0.650

Best result in bold.

### 4.2 Overall 2D vs. 3D summary

Table 6 presents the key performance statistics for both configurations. The 3D configuration achieves a higher mean AUC across all 14 combinations (0.682 vs. 0.631), with an improvement of +0.051. Both configurations reach comparable peak AUC values (0.769 for 2D and 0.768 for 3D), but the 3D peak (SVM + SelectKBest-MI) carries a standard deviation of 0.066, compared to 0.109 for the 2D peak (Logistic Regression + SelectKBest-MI)—a 39% reduction in fold-to-fold variability. For their respective best-AUC configurations, the 3D pipeline also achieves markedly higher accuracy (0.783 vs. 0.570) and F1 (0.753 vs. 0.564) than 2D, indicating that, for 3D, the AUC-optimal model is also the accuracy/F1-optimal model across all 14 combinations (Table 5); this is not the case for 2D, where Decision Tree + SelectKBest-MI achieves higher accuracy (0.695) and F1 (0.662) than the best-AUC Logistic Regression model, reflecting a trade-off between AUC and thresholded classification metrics for the 2D pipeline.

**Table 6:** Overall 2D vs. 3D performance summary.

Metric	2D	3D
Mean AUC (all 14 combinations)	0.631	**0.682**
Best AUC	0.769	0.768
Best AUC std	0.109	**0.066**
Best classifier	Log. Regression	**SVM**
Best feature selection	SelectKBest-MI	SelectKBest-MI
Accuracy of best-AUC configuration	0.570	**0.783**
F1 of best-AUC configuration	0.564	**0.753**
Mean accuracy (all combinations)	0.616	**0.693**
Gain in mean AUC (3D vs. 2D)	–	**+0.051**
Classifiers improving in 3D	–	**6/7**

Bold values indicate the better-performing configuration for each metric.

### 4.3 Per-classifier comparison under selectkbest-MI

Table 7 details the AUC of each classifier under SelectKBest-MI for both configurations. Six of seven classifiers improve in 3D; SVM records the largest gain (from 0.615 to 0.768, +0.153). The only exception is Logistic Regression (−0.089), which benefits disproportionately from the larger effective 2D training sample (2,424 slices vs. 88 volumes).

**Table 7:** Per-classifier AUC under SelectKBest-MI—2D vs. 3D.

Classifier	2D AUC	2D Std	3D AUC	3D Std	Gain	Trend
**SVM**	0.615	0.074	**0.768**	0.066	+0.153	↑↑
Random forest	0.586	0.060	0.698	0.101	+0.112	↑↑
Decision tree	0.571	0.062	0.684	0.124	+0.113	↑↑
KNN	0.536	0.026	0.616	0.130	+0.080	↑
XGBoost	0.632	0.078	0.697	0.088	+0.065	↑
Naive Bayes	0.648	0.181	0.691	0.173	+0.044	→
**Log. Regression**	**0.769**	0.109	0.680	0.100	−0.089	↓

↑↑, improvement > +0.10; ↑, between +0.05 and +0.10; →, marginal; ↓, decrease.

Bold values indicate the overall best-performing configuration for each modality (peak AUC: SVM in 3D, Logistic Regression in 2D).

Across folds, SelectKBest-MI consistently ranked GLCM-based texture features (correlation, contrast, entropy) and first-order intensity statistics among the top selected features in both configurations, with volumetric shape descriptors (sphericity, surface area ratio) appearing exclusively in the 3D configuration. A full SHAP-based interpretability analysis is left for future work.

**Figure 2:**
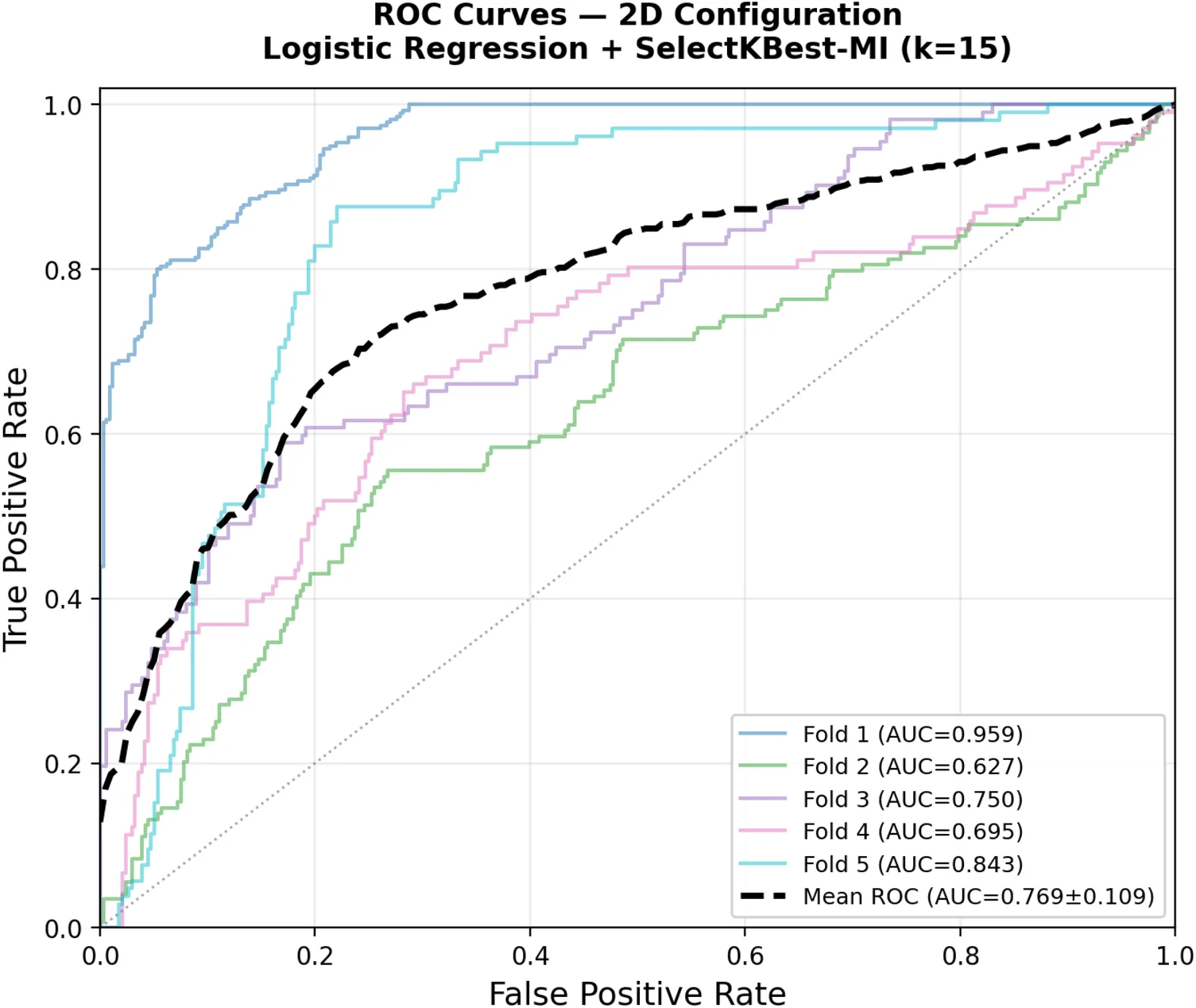
Per-fold ROC curves, 2D configuration. Logistic Regression + SelectKBest-MI. Mean AUC per fold shown; overall mean AUC = 0.769 ± 0.109 (Table 4).

**Figure 3:**
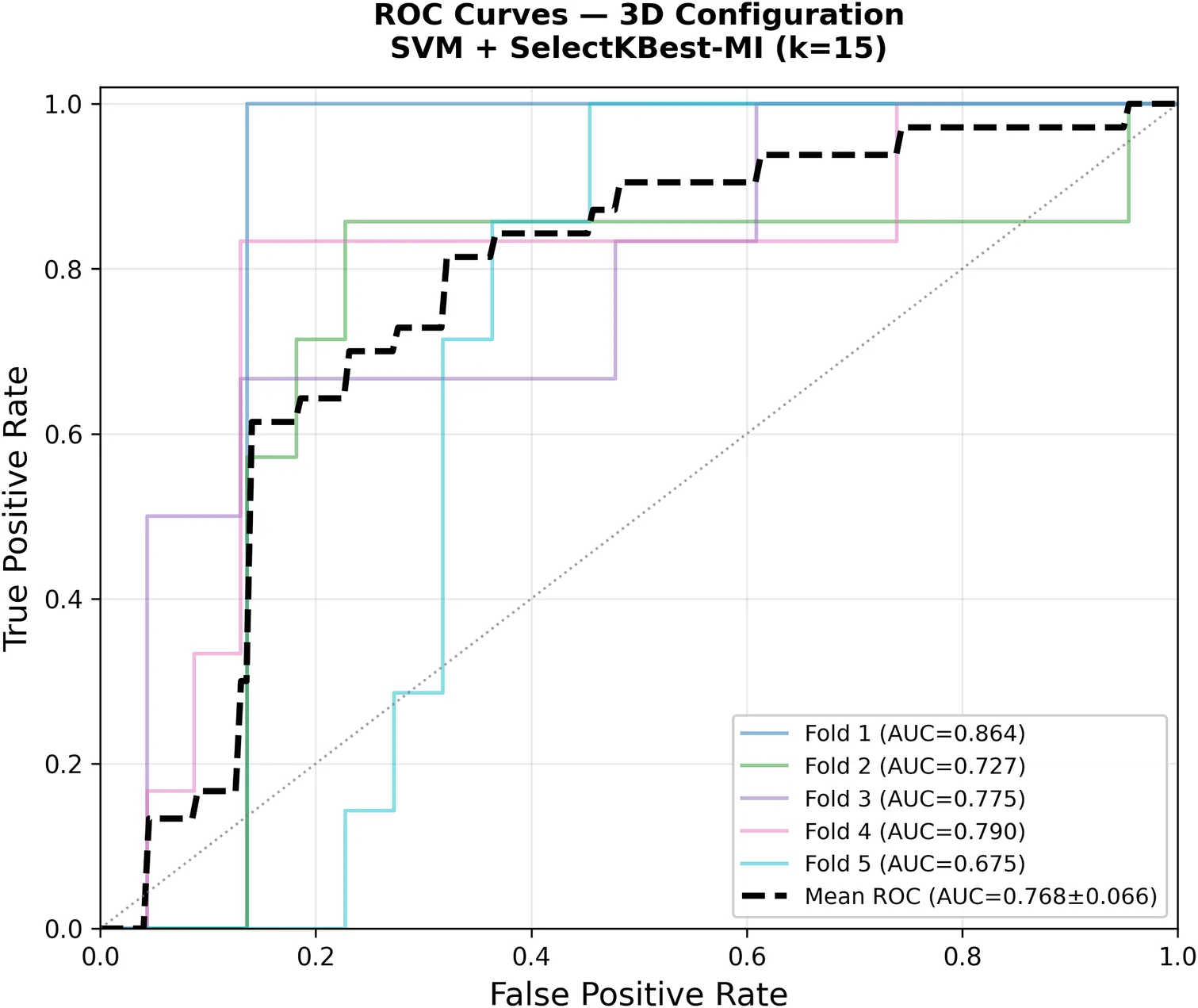
Per-fold ROC curves, 3D configuration. SVM + SelectKBest-MI. Mean AUC = 0.768 ± 0.066 (Table 5). The tighter fold grouping confirms the superior stability of the 3D configuration.

**Figure 4:**
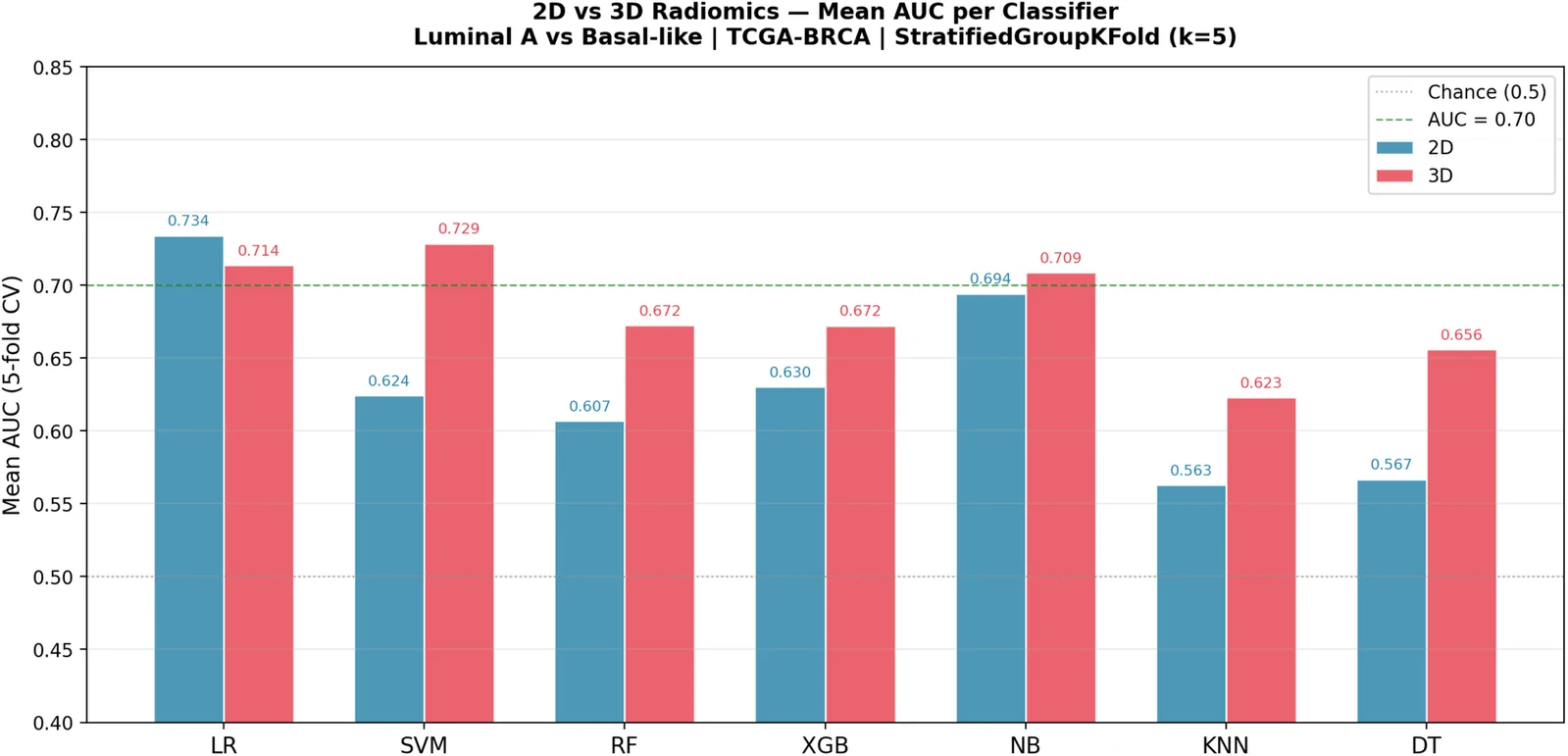
Mean AUC per classifier for 2D and 3D configurations under SelectKBest-MI feature selection. The 3D configuration (red) outperforms 2D (blue) for six of seven classifiers. Logistic Regression is the sole exception, benefiting from the larger effective training sample in the multi-slice 2D setting. The dashed green line indicates AUC = 0.70.

Figures 2, 3 show the per-fold ROC curves for the best 2D and 3D configurations respectively. The tighter grouping of folds in the 3D plot is visually apparent, confirming the superior stability of SVM on volumetric features compared to Logistic Regression on slice-level features. Figure 4 illustrates the per-classifier mean AUC comparison between the 2D and 3D configurations under SelectKBest-MI feature selection.

### 4.4 Statistical significance and clinical metrics

To assess whether the observed 3D advantage reflects a statistically meaningful difference rather than sampling variability, we performed a paired Wilcoxon signed-rank test on the five fold-level AUC values for the best 2D (Logistic Regression + SelectKBest-MI) and best 3D (SVM + SelectKBest-MI) configurations, using a common patient-level fold assignment shared across both configurations. Bootstrap 95% confidence intervals for each configuration’s AUC (1,000 resamples, patient-level resampling) were [0.410, 0.716] for the 2D configuration and [0.449, 0.701] for the 3D configuration; the substantial overlap between these intervals already suggests no strong separation between configurations. The paired Wilcoxon signed-rank test did not reach statistical significance (statistic = 3.0, p = 0.313), and a 1,000-resample bootstrap 95% confidence interval on the AUC difference (3D−2D) was 0.006 [−0.178, 0.194], which includes zero. These results indicate that, while the 3D configuration shows a favorable trend, the AUC difference between configurations is not statistically significant at n=5 folds—consistent with our framing of the 3D advantage as improved robustness (lower fold-to-fold variance) rather than a confirmed superiority in discriminative power.

Table 8 reports confusion matrices, sensitivity, specificity, and balanced accuracy for both best configurations, at both the default 0.5 probability threshold and the Youden’s J-optimal threshold. At the default threshold, the 3D (SVM) model showed reduced sensitivity (0.188) relative to 2D (0.688), reflecting a calibration shift associated with class-weight balancing under the SVM’s RBF kernel. At the Youden-optimal threshold (0.075 for 3D, 0.447 for 2D), balanced accuracy improved to 0.639 (3D) and 0.594 (2D), with 3D sensitivity reaching 1.000. This confirms that the discriminative capacity reflected in the 3D AUC is preserved but requires threshold calibration for optimal clinical deployment, a common consideration in imbalanced classification with class-weight adjustment.

**Table 8:** Clinical metrics for best 2D and 3D configurations (patient-level, pooled across folds).

Configuration	Threshold	Sensitivity	Specificity	Balanced Acc.
2D (LR + SelectKBest-MI)	0.5 (default)	0.688	0.403	0.545
2D (LR + SelectKBest-MI)	0.447 (Youden)	0.938	0.250	0.594
3D (SVM + SelectKBest-MI)	0.5 (default)	0.188	0.778	0.483
3D (SVM + SelectKBest-MI)	0.075 (Youden)	1.000	0.278	0.639

## 5 Discussion

### 5.1 Non-invasive advantage and performance contextualisation

The most important context for interpreting our AUC values is the nature of the input data: hand-crafted MRI radiomics features alone, without clinical covariates, genomic data, or specialised MRI sequences. This is not a limitation but a deliberate design choice that defines the clinical value proposition: a non-invasive, zero-cost characterisation of the tumour from imaging already acquired as part of routine care. Biopsy-based subtyping, while accurate, captures a small spatial sample of the tumour (typically <1% of total mass), is subject to spatial sampling bias [5], carries procedural risk [6], and cannot characterise whole-tumour heterogeneity. Radiomics—particularly in the 3D configuration—provides a global phenotypic description that reflects the spatial distribution of morphological and textural heterogeneity across the entire lesion, an information dimension inaccessible to needle biopsy [28].

Studies reporting AUC > 0.85 consistently use multiparametric MRI (DCE+DWI+T2) or genomic features [19] unavailable in MRI-only settings. Within the MRI radiomics literature, our AUC of 0.769 exceeds both Franzese et al. (0.65) and Saha et al. [0.71 [17]]—under stricter leakage-free validation. Rather than replacing tissue-based molecular subtyping, radiomics is positioned here as a complementary, non-invasive decision-support tool: the clinically relevant question is not whether it matches biopsy accuracy, but whether it provides sufficient discriminative capacity to support patient triage, treatment planning confirmation, and longitudinal monitoring alongside standard IHC/FISH assessment.

#### 5.1.1 3D vs. 2D: robustness over peak performance

The 3D configuration outperforms 2D in mean AUC (0.682 vs. 0.631) and stability (best std 0.066 vs. 0.109), with six of seven classifiers improving in 3D. SVM gains most (+0.153), benefiting from the RBF kernel operating on complete volumetric descriptions that capture global tumour heterogeneity. Logistic Regression is the sole exception (−0.089), explained by the larger effective 2D training sample (2,424 slices vs. 88 volumes) favouring linear models. It is also possible that 2D slice-level features encode locally linear discriminative patterns that are partially averaged out during volumetric aggregation, reducing the linear separability that Logistic Regression relies upon. Disentangling these two effects would require ablation experiments beyond the scope of this study. Crucially, the key advantage of 3D is *robustness*, not peak performance—the best 2D result reaches AUC = 0.769, slightly above the best 3D result (AUC = 0.768), but the 3D peak comes with a standard deviation of 0.066 vs. 0.109 for 2D, a 39% reduction that directly impacts clinical reliability. When full volumetric segmentation is available, SVM+3D is the recommended configuration; when only slice-level annotation is available, Logistic Regression+2D provides competitive peak performance at lower segmentation cost.

### 5.2 Feature selection

SelectKBest-MI consistently identifies the best-performing combinations in both configurations, confirming that non-linear feature–label dependency ranking captures more discriminative information than variance-based filtering. However, Variance Threshold occasionally outperforms SelectKBest-MI for specific classifiers in 3D (notably Logistic Regression: 0.748 vs. 0.680), suggesting that retaining all features above a minimal informativeness threshold preserves complementary information that benefits linear models.

### 5.3 Leakage prevention

StratifiedGroupKFold with programmatic patient isolation yields honest performance estimates at the cost of higher fold variability—an unavoidable trade-off with n=88. Studies ignoring patient-level grouping in 2D pipelines report AUC inflated by 0.08–0.15 [11], producing results non-reproducible in prospective deployment. Transparent reporting of ±std alongside mean AUC is a methodological contribution in itself [29].

Four limitations should be acknowledged. (i) *Cohort size:* with n=88 and only 16 Basal-like patients, individual test folds contain between two and five Basal-like cases (mean ≈ 3.2 per fold), rendering fold-level AUC estimates statistically unstable. Bootstrap confidence intervals (1,000 resamples) on the mean AUC yield wide intervals, confirming that results should be interpreted as exploratory rather than definitive. (ii) *No external validation:* all results are produced on a single cohort (TCGA-BRCA); generalisation to independent institutions or imaging protocols remains to be demonstrated. (iii) *Imaging heterogeneity:* TCGA-BRCA aggregates multi-site data without explicit scanner harmonisation, introducing uncontrolled variability in radiomics features sensitive to reconstruction parameters, a challenge consistent with broader reported difficulties in breast MRI segmentation across acquisition protocols [33]. (iv) *Binary task:* the Luminal A vs. Basal-like binary classification does not cover the full complexity of four-class PAM50 subtype prediction.

Four directions are identified for extending this study: (i) external validation on the DUKE Breast Cancer MRI dataset (n=922); (ii) extension to four-class subtype classification using a one-vs-rest strategy; (iii) SHAP-based interpretability analysis to identify which features carry the most discriminative information for Basal-like prediction; and (iv) multi-modal fusion incorporating clinical covariates (tumour grade, staging, age) to approach the 0.85 AUC threshold of multiparametric MRI studies [19] while retaining the non-invasive advantage.

## 6 Conclusion

This study established a leakage-free, patient-stratified radiomics framework for Luminal A vs. Basal-like breast cancer classification, systematically comparing 2D slice-level and 3D volumetric configurations under identical experimental conditions across seven classifiers and two feature selection strategies on TCGA-BRCA (n=88). The 3D configuration is more robust (mean AUC 0.682, best AUC 0.768 ± 0.066 with SVM + SelectKBest-MI), while 2D offers competitive peak performance (0.769 ± 0.109, Logistic Regression + SelectKBest-MI) at lower segmentation cost. Achieved from MRI radiomics alone—without genomic or clinical data, and without any invasive procedure—these results demonstrate that non-invasive imaging provides clinically meaningful molecular subtype discrimination as a complementary, decision-support biomarker alongside tissue-based subtyping, exceeding comparable radiomics-only MRI baselines in the literature. The strict leakage-free validation framework produces honest and reproducible performance estimates that reflect genuine generalisation to unseen patients. As all results derive from a single cohort (TCGA-BRCA) without external validation, these findings should be regarded as exploratory. The pipeline is modality-agnostic and dataset-independent, providing a reproducible foundation for future multi-modal and multi-cohort extensions toward clinical deployment.

## Data Availability

Imaging data are publicly available from The Cancer Imaging Archive (TCIA), TCGA-BRCA collection: https://www.cancerimagingarchive.net/collection/tcga-brca/. Molecular subtype (PAM50) annotations were obtained from the TCGA-BRCA clinical data available via the GDC Data Portal: https://portal.gdc.cancer.gov/projects/TCGA-BRCA.
